# Aneurism

**Published:** 1895-03-16

**Authors:** A. Pearce Gould

**Affiliations:** Senior Assistant Surgeon to the Middlesex Hospital


### Aneurism.

By A. Pearce Gould, F.R.C.S., Senior Assistant Surgeon to the Middlesex Hospital.

To the question, "What is an aneurism?" many answers have been given. This is chiefly due to the fact that many conditions, differing widely and essentially, have had this term applied to them, and the definitions given have been attempts to cover all these various forms of disease. But in a definition, as in much else, simplicity is of the utmost importance, and happily we can give a very simple one of the term aneurism. By an aneurism should be understood "a blood-tumour communicating with an artery." It is worth while just to look at the various points of this definition, and notice what it includes and excludes, and also the importance of the facts it states. First of all, an aneurism is a tumour: it will often save an error in diagnosis to remember that "no tumour, no aneurism." A freely-pulsating abdominal aorta, an abnormally-placed artery, a slightly enlarged and prominently-pulsating artery—either of these conditions is often mistaken for an aneurism, and the mistake can always be avoided if the medical attendant takes pains to verify the existence of a distinct tumour before he diagnoses aneurism. By the word tumour we mean not only a swelling, but a fairly circumscribed swelling, and so by our definition we at once exclude mere extravasations of blood in the cellular tissue of a part, however extensive. It is important to insist upon this point, for only circumscribed collections of blood can be called aneurisms. To put an extreme case: If an artery is wounded, and pours blood into the peritoneal cavity, we should not think of calling such a bloody swelling an aneurism; and just the same if a wounded artery pours blood into the retro-peritoneal tissue and forms a great collection there, it is not an aneurism. This idea of the distinct limitation of the blood by a sac or wall is essential to the correct definition of an aneurism, and by losing sight of it serious errors have crept into surgical practice.

The second statement in our definition—that the tumour is a blood tumour—requires but a few words. This is obvious. The tumour not only contains blood, but is formed practically entirely of blood, and when it has ceased to be a blood tumour it has ceased to be an aneurism.

It is the third point in our definition, however, that is the most essential—the statement that the blood-tumour communicates with an artery. By this communication is meant a large, free, gross communication, such a communication as permits of the blood flowing through the aneurism more or less freely. This at once excludes two very important classes of blood-tumours—hæmatoma and blood-cysts. A hæmatoma is a primary blood-tumour, almost always traumatic in origin, which has no gross palpable communication with any artery or vein, and which certainly is not the seat of a flow of blood through it. As compared with aneurism, it is an entirely passive condition, the result of a hæmorrhage, but of a hæmorrhage that has ceased, and that does not affect gravely the circulation in the limbs. A blood-cyst is a very soft sarcomatous growth into which hæmorrhage has occurred. The hæmorrhage is a secondary stage of the local trouble, and has escaped from the very thin-walled, delicate vessels to be found in these growths. Such a condition is absolutely unlike an aneurism, and should never be confused with it. The communication with an artery is the prime fact about an aneurism; it is this which explains the great clinical features of an aneurism, and when we come to speak of the treatment of aneurism, we shall find that the cure of an aneurism is only effected when the communication of the tumour with the artery is obliterated, and the aim of all treatment is to secure this obliteration with the smallest possible disturbance of the parts or injury to the patient.

Our definition excludes from the category of aneurisms cases of what are known as arterial varix, and aneurism by anastomosis, and also all cases of subcutaneous rupture of arteries. These have been included by some authorities, but they differ in nature altogether from typical aneurisms, and they demand wholly different treatment, and on this account particularly it is most important that they should be kept quite distinct, and not included in the same category in any systematic classification. The case that presents most difficulty is that of simple dilatation of an artery due to the yielding of a diseased artery to the pressure of the blood. From one point of view, such an enlargement can hardly be called "a blood tumour communicating with an artery," for it is an artery itself. But in the aorta particularly, such dilatations are liable to assume great proportions, and cause most serious symptoms simply from their size and pressure; they are, indeed, the most frequent form of mediastinal tumour. And so by common consent they are always included among aneurisms, and it is all the more needful to do so as such a dilatation of an artery is often associated with and leads on to a pouching of the arterial wall, and the development of a typical sacculated aneurism. If we seek for analogies in other organs we do not get much help, it is true. A dilated bronchus is never called an air-tumour, or a bronchial tumour; a dilated stomach is never spoken of as a tumour of the stomach; but a dilated coil of intestine blocked with fœces is called a fœcal tumour. A dilated vein is a varix, and is only called a venous tumour under very special circumstances, when there is a great and very localised bulging of a vein forming a more or less globular swelling. Unquestionably the facts that fusiform aneurism results from the yielding of the wall of an artery to the pressure of the blood within it, and that it possesses a distinct limiting sac surrounding the blood, are the main reasons for grouping this condition with typical aneurisms. How aneurisms arise, and how they should be classified, will form the subject of the next paper.

---

The hotwell springs at Bristol were formerly highly favoured by the public and medical authorities. Local enterprise and the golden sinews of Mr. Newnes, M.P., have recently drawn attention to these baths; and the new Clifton Spa and Hydro, with its grand pump room, were recently opened to the public.

